# Tetraspanins, GLAST and L1CAM Quantification in Single Extracellular Vesicles from Cerebrospinal Fluid and Serum of People with Multiple Sclerosis

**DOI:** 10.3390/biomedicines12102245

**Published:** 2024-10-02

**Authors:** Rocío Del Carmen Bravo-Miana, Jone Karmele Arizaga-Echebarria, Valeria Sabas-Ortega, Hirune Crespillo-Velasco, Alvaro Prada, Tamara Castillo-Triviño, David Otaegui

**Affiliations:** 1 Neuroimmunology Group, Neuroscience Area, Biogipuzkoa Health Research Institute, 20014 San Sebastián, Spain; jonekarmele.arizagaechebarria@bio-gipuzkoa.eus (J.K.A.-E.); vsabasorteg@alumni.unav.es (V.S.-O.); hirune.crespillovelasco@bio-gipuzkoa.eus (H.C.-V.); alvarojose.pradainurrategui@osakidetza.eus (A.P.); tamara.castillotrivino@osakidetza.eus (T.C.-T.); 2 Centro de Investigación Biomédica en Red de Enfermedades Neurodegenerativas, Instituto de Salud Carlos III, 28029 Madrid, Spain; 3 Immunology Department, Donostia University Hospital, 20014 San Sebastián, Spain; 4 Neurology Department, Donostia University Hospital, 20014 San Sebastián, Spain

**Keywords:** extracellular vesicles, multiple sclerosis disease, cerebrospinal fluid-derived EVs, serum-derived EVs, tetraspanins, GLAST, L1CAM, central nervous system-derived EVs, neural-derived EVs, brain-derived EVs

## Abstract

**Objective:** This study aimed to unravel the single tetraspanin pattern of extracellular vesicles (EVs), L1CAM^+^ and GLAST^+^ EV levels as diagnostic biomarkers to stratify people with multiple sclerosis (pwMS), specifically relapsing–remitting (RRMS) and primary progressive (PPMS). **Methods:** The ExoView platform was used to directly track single EVs using a clinically feasible volume of cerebrospinal fluid (CSF) and serum samples. This technology allowed us to examine the patterns of classical tetraspanin and quantify the levels of L1CAM and GLAST proteins, commonly used to immunoisolate putative neuron- and astrocyte-derived EVs. **Results:** The tetraspanin EV pattern does not allow us to differentiate RRMS, PPMS and non-MS donors neither in CSF nor serum, but this was associated with the type of biofluid. L1CAM^+^ and GLAST^+^ EVs showed a very low presence of tetraspanin proteins. Additionally, a significant decrease in the particle count of L1CAM^+^ EVs was detected in L1CAM-captured spots, and L1CAM^+^ and GLAST^+^ EVs decreased in GLAST-captured spots in the CSF from PPMS subjects compared to RRMS. Interestingly, only GLAST^+^ EVs exhibited a lower quantity in the CSF from PPMS compared to both MS and non-MS samples. Finally, GLAST^+^ EVs demonstrated a medium negative and significative correlation with GFAP levels—a biomarker of MS progression, astrocyte damage and neurodegenerative processes. **Conclusions:** ExoView technology could track neural EV biomarkers and be potentially useful in the diagnostic evaluation and follow-up of pwMS. GLAST^+^ EVs might provide insights into the etiology of PPMS and could offer small windows to elucidate the molecular mechanisms behind its clinical presentation.

## 1. Introduction

Multiple sclerosis (MS) is a chronic immunomediated and neurodegenerative condition in which environmental and susceptible genetic factors are associated with the disease pathogenesis, although its triggering cause remains unclear. Even if autoimmunity plays a major role in the pathogenesis, an accumulative process of remyelination failure, axonal damage, and neuronal loss drives the progression and neurodegeneration [1,2,3]. Basically, its clinical course is characterized by three major MS phenotypes, known as primary progressive MS (PPMS), relapsing–remitting MS (RRMS), and secondary progressive MS [4]. Diagnosis and patient stratification among the highly variable and heterogeneous clinical MS phenotypes have a critical impact on therapeutic decisions. Nowadays, clinical practice is mainly supported by magnetic resonance imaging and the pattern of oligoclonal bands (OCBs) in cerebrospinal fluid (CSF) and serum [5]; however, they are not able to deeply explore the molecular-level information. In this scenario, the use of extracellular vesicles (EVs) as the next generation of neural circulating biomarkers has been proposed [6].

EVs are lipid bilayer membrane-delimited nanoparticles whose bioactive cargo reflects the molecular state of their cells of origin. Considering circulating EVs in biofluids, such as CSF and blood, as small windows into the brain of people with different neurological diseases, like MS, is a well-established idea [7,8]. Nevertheless, the use of EVs as biomarkers has not reached pre-clinical and clinical studies, probably because of the complex and time-consuming EV isolation process and the lack of a gold-standard isolation method. Single-particle interferometric reflectance imaging sensor (SP-IRIS)-based ExoView R200+ technology could address these challenges by enabling the phenotyping analysis of unprocessed samples, avoiding the EV isolation, with a clinically feasible sample volume [9,10]. CD63, CD81, and CD9, members of the tetraspanin family, are the most commonly reported transmembrane EV proteins and are widely used in immunoisolation techniques. However, it is recognized that not all EVs present tetraspanins EV proteins. Therefore, there are no universal EV markers nor clearly accepted markers to distinguish EV subpopulations [11]. Although still controversial, the principal advances in the translational neuroscience field were made using the L1 cell adhesion molecule (L1CAM): L1CAM^+^ EVs [6]. This protein is being extensively discussed due to its tissue specificity [12,13]. In MS, L1CAM and the glutamate aspartate transporter (GLAST) were proteins used to immunoisolate putative neuron- and astrocytes-derived EVs, respectively, from the plasma of people with MS (pwMS), analyzing their specific cargo [14]. Nonetheless, they did not quantify single L1CAM^+^ and GLAST^+^ EVs per se in unprocessed CSF or serum samples. To date, no previous studies have examined the single EV tetraspanin pattern, L1CAM^+^, and GLAST^+^ EV levels as a way to stratify pwMS. The aim of this study was to unravel the single tetraspanins, L1CAM^+^ and GLAST^+^ EVs in CSF and serum diagnostic samples from RRMS and PPMS using SP-IRIS-based ExoView R200+ technology.

## 2. Materials and Methods

### 2.1. CSF and Serum Paired Samples

The pwMS selection (10 RRMS and 9 PPMS samples) was based on McDonald criteria [15], including the presence of a positive OCB-IgG pattern. Paired CSF and serum samples were obtained via lumbar puncture and subsequent venipuncture for diagnostic purposes, and the remaining aliquots were collected, processed, and immediately stored at −80 °C in the Basque Biobank (BIOEF, Donostia University Hospital, San Sebastián, Spain). Due to the impossibility of obtaining CSF from healthy controls, we used a group of 10 non-MS samples that did not present the OCB-IgG pattern nor a profile in the hospital’s MS database. This retrospective study was approved by the Ethics Committee of Euskadi (DOB-EVS-2023-01) on 17 March 2023, following the Declaration of Helsinki, with the need for written informed consent. Age, gender, type of disease, and the clinical expanded disability status scale (EDSS score) [16] were retrieved from each patient. Table 1 provides a summary of sample characteristics. RRMS and PPMS diagnostic samples were sex-matched across the groups but, as expected in the MS pathology [17], the age of the PPMS group is higher (although not statistically). Before being used, samples were centrifuged at 2500× *g* for 15 min at 4 °C.

### 2.2. Human Tetraspanin Assay

A total of 50 µL of the incubation solution (1×)-diluted CSF (1/10) and serum (1/100) was incubated onto human tetraspanin chips (Leprechaun, Unchained Labs, Brighton, MA, USA), previously prescanned using the ExoView scanner 3.2.1 in the SP-IRIS-based ExoView R200+ platform (NanoView Biosciences, Brighton, MA, USA). CD63 (clone H5C6), CD81 (clone JS-81), CD9 (clone HI9a), and Mouse IgG (clone MOPC-21) capture antibodies were printed onto the chip by default. Specifically for serum or plasma, the chips included the CD41a platelet marker (clone HIP8) as a capture antibody (human tetraspanin plasma kit, Leprechaun, Unchained Labs, Brighton, MA, USA). In this assay, fluorescently labelled antibodies (detection antibodies) targeting the same epitopes were used following the manufacturer’s recommended dilution, as well as the exosome protocol of the automatic chip washer (Unchained Labs, Brighton, MA, USA). Then, the chips were dried for 30 min at room temperature (RT) and subsequently scanned. All results represent the mean of triplicate spots, serving as technical replicates within the same analyzed sample. As a negative control, CSF or serum samples were disrupted with 1% triton X-100 (VWR Chemicals, Mississauga, ON, Canada) for 30 min at RT. Then, with the purpose of depleting the disrupted membranes, the samples were centrifuged at 10,000× *g* for 15 min before supernatant incubation onto the chip (as recommended in [11]). Only fluorescent events, when intact EV samples presented a tendency or a significant increase compared to disrupted EV samples, were considered for further analysis.

### 2.3. Human Tetraspanin Flex Assay

In addition to the CD63-, CD81-, CD9-, and IgG-captured antibodies, the flex chips presented Flex1- and Flex2-captured antibodies that have specific junctions with linker1 and 2. Flex1 and 2 can be functionalized with custom antibodies of choice previously conjugated with the linkers. In this case, linker1 and linker2 were conjugated with L1CAM clone 5G3 (BD Biosciences, San Jose, CA, EEUU, #554273) and GLAST clone EPR12686 (Abcam, Cambridge, UK, #ab240235), respectively, following the kit instructions. These two antibodies recognize external epitopes to capture intact EVs. Then, chips were customized using the flex prep protocol in the chip washer (Unchained Labs, Brighton, MA, USA). Moreover, 50 µL of the incubation solution (1×)-diluted CSF (1/10) and serum (1/1000) were incubated onto the chips, followed by the protocol used for the tetraspanin kit mentioned above. In this case, 3 µL (2.5 µg/mL) of L1CAM, 0.75 µL (1.25 µg/mL) of GLAST, and 0.6 µL (1 µg/mL) of CD9 (CF^®^ 488A) were used for each flex chip as detection antibodies. L1CAM and GLAST were previously labelled using Alexa Fluor^®^ 647 (ab269823) and Alexa Fluor^®^ 555 (ab269820) conjugation kits (Abcam, Cambridge, UK), respectively, following the kit procedure. The same negative controls of CSF- or serum-disrupted EV samples were used.

### 2.4. Data Processing

Data obtained from prescanning and scanning the chips were uploaded to the ExoView analyzer software 3.2 (NanoView Biosciences, Brighton, MA, USA) to perform a quality control of the chips. Data were adjusted by dilution factor. Spots that had been affected during incubation, labelling, or washing were excluded. Finally, specific fluorescent intensity (arbitrary units) cut-off values for each detection antibody were defined using the isotype control (IgG) spots. All selected cut-off values enabled up to an average of 10% IgG signals being included in the isotype control spots. For the tetraspanin chips, the cut-off values were set at 300, 300, and 500 for CD63, CD81, and CD9, respectively. In the case of flex tetraspanin chips, the cut-off values were 500, 500, and 500 for L1CAM, GLAST, and CD9, respectively. Positive fluorescent events in the isotype control (IgG), upon the cut-off correction, are considered as the basal background. The data for all capture spots were normalized across different chips by background subtraction. Individual data files from each sample, with the total number of particles and their colocalization patterns in all captured spots (in triplicate), were analyzed using Python v3.11.0 scripts (Python Software Foundation, https://www.python.org/, accessed on 4 September 2024). Custom scripts developed in-house enabled the automated analysis of unlimited individual sample data, streamlining data management, generating heatmaps, and conducting statistical and hierarchical clustering analysis.

### 2.5. Measurement of Neurofilament Light and Glial Fibrillary Acidic Protein Levels in CSF

Neurofilament light (NfL) chain and glial fibrillary acidic protein (GFAP) levels were measured in CSF using the commercial Ella™ microfluidic platform (Bio-Techne, Minneapolis, MN, USA) following the kit instructions. Briefly, diluted CSF (1/2) was incubated in the SimplePlex^®^ human NfL assay cartridge (#SPCKB-PS-002448) and in the SimplePlex human GFAP (2nd Gen) assay Cartridge (#SPCKB-PS-009134). Then, 1 mL of the wash buffer was added to the specific wells, and 50 µL of the high and low concentration of NfL/GFAP was used as the internal control. The average of the NfL/GFAP triplicate (measured in pg/mL) obtained for each sample was used in correlation analysis.

### 2.6. Statistical Analysis

The results were expressed as median ± range or median ± interquartile range (IQR). Statistical significance was determined using a parametric or non-parametric ANOVA or unpaired *t*-test, performed with Graph-Pad Prism 8.0.1 (GraphPad Prism Software) and Python. The Spearman’s rank correlation coefficient test was used for the correlation between continuous variables. Differences with *p* < 0.05 were considered statistically significant.

## 3. Results

### 3.1. Tetraspanin Pattern Characterization in CSF and Serum EVs from pwMS

**Figure 1:**
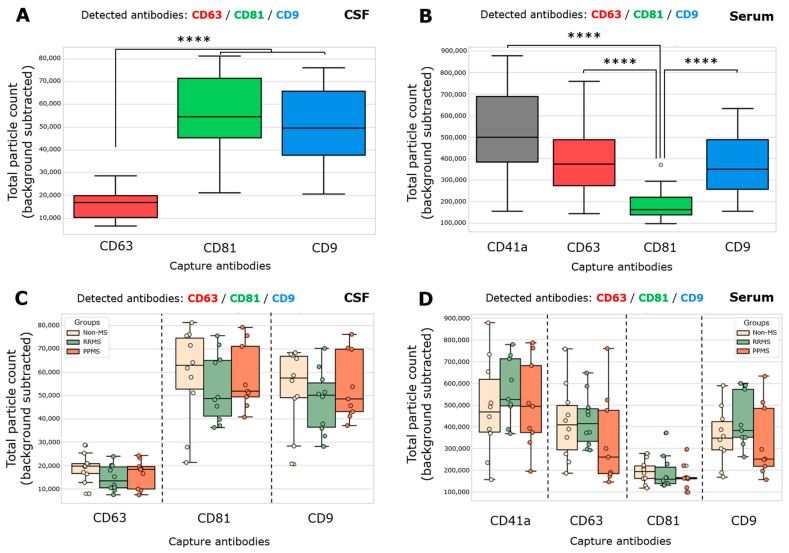
Both MS and non-MS samples exhibited significantly lower enrichment of CD63^+^ EVs in CSF and CD81^+^ EVs in serum. (**A**,**C**) Total single EV-captured (CD63-spots, CD81-spots, CD9-spots) measured in triplicate from CSF samples by ExoView R200+ technology. (**A**) A significant increase in the total particle count of CD81^+^ EVs and CD9^+^ EVs respect to CD63^+^ EVs was observed (**** *p* < 0.0001; ordinary one-way ANOVA test, Tukey’s post test). (**B**,**D**) Total single EV-captured (CD41a-spots, CD63-spots, CD81-spots, CD9-spots) measured in triplicate from serum samples. (**B**) A significant increase in the total particle count of CD41a^+^ EVs, CD63^+^ EVs, and CD9^+^ EVs respect to CD81^+^ EVs was observed (**** *p* < 0.0001; Kruskal–Wallis test, Dunn’s post test). The total particle count of the isotype control (IgG) was subtracted from the total particle count on each capture spot. Data are expressed as the median ± range of at least nine independent determinations by ExoView R200+ technology.

Total particle counts of CD63-, CD81-, and CD9-spots in CSF and CD41a-, CD63-, CD81-, and CD9-spots in serum was determined from both non-MS and MS samples. The total particle count in each spot is the number of all EVs that present one or more fluorescent detection proteins. Considering the CSF, CD81^+^ EVs and CD9^+^ EVs were significantly higher than CD63^+^ EVs (Figure 1A). In serum, a gradient from a high to a low quantity of CD41a^+^ > CD63^+^/CD9^+^ > CD81^+^ EVs was found (Figure 1B). Then, the total particle count for each capture antibody was contrasted among the groups in both biofluids, and no significant differences were registered (Figure 1C,D). In serum, the total particle count of CD63- and CD9-captured spots incubated with RRMS showed a trend toward increasing compared to PPMS, but without statistical significance (Figure 1D). As expected, disrupted EV spots exhibited a negligible positive signal compared to spots incubated with intact EV samples (Figure S1).

### 3.2. The Tetraspanin Pattern Does Not Allow to Differentiate RRMS, PPMS and Non-MS Donors Neither in CSF nor Serum

**Figure 2:**
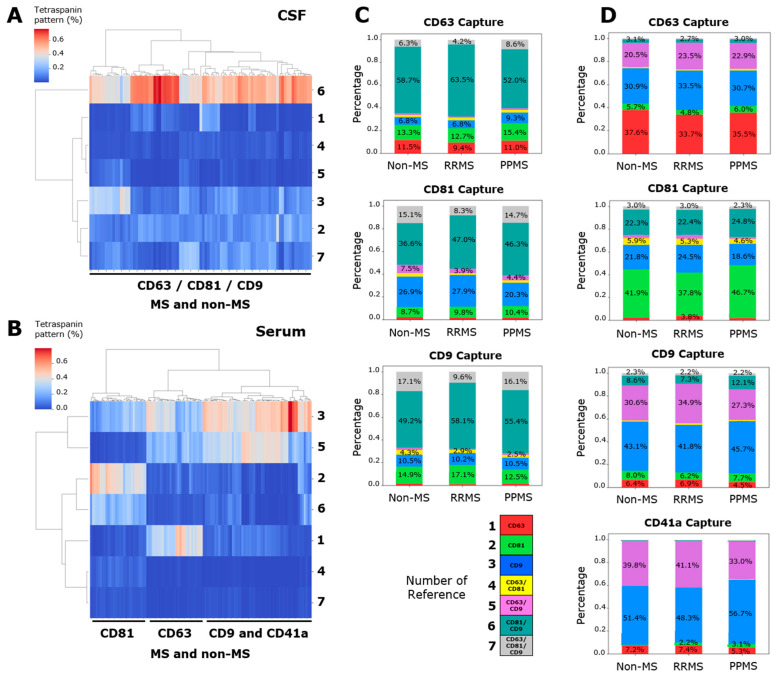
CSF showed a particularly higher percentage of double-positive CD81^+^/CD9^+^ EVs in all captured spots. Unsupervised hierarchical clustering of the tetraspanin pattern in (**A**) CSF and (**B**) serum. Stacked bars of (**C**) CD63-, CD81-, and CD9-captured spots in CSF and (**D**) CD41a, CD63-, CD81-, and CD9-captured spots in serum. These charts were utilized to represent the average percentage of this pattern in each sample. (**A**,**C**) Although the tetraspanin pattern does not segregate RRMS, PPMS and non-MS samples, a particularly high percentage of double-positive CD81^+^/CD9^+^ EVs was evidenced in CSF. (**B**,**D**) In serum, a high percentage of double-positive CD63^+^/CD9^+^ EVs was evidenced, except for CD81-captured spots. Additionally, the tetraspanin pattern of CD9- and CD41a-captured spots were closely related. Data are expressed as the percentage (heatmap) or the mean of percentage (stacked bars) of at least nine independent determinations by ExoView R200+ technology.

In addition to the total particle count present in each spot, the ExoView technology allows for the obtention of a pattern of single- (CD63^+^, CD81^+^, and CD9^+^), double- (CD63^+^/CD81^+^, CD63^+^/CD9^+^, and CD81^+^/CD9^+^) and triple- (CD63^+^/CD81^+^/CD9^+^) positive signals in each single EV. This tetraspanin colocalization pattern could be unique for each sample, and it could be used to analyze the performance of these proteins to segregate different patient samples using clustering techniques. An unsupervised hierarchical clustering was employed, based on the percentage of the seven signals with respect to the total count. This pattern does not differentiate among RRMS, PPMS and non-MS donors in CSF (Figure 2A) nor serum (Figure 2B). However, a segregation between CD41a/CD9 spots regarding the CD81 and CD63 spots was evidenced (Figure 2B), with CD41a and CD9 being similarly related, as was previously published [18]. Interestingly, a notably high percentage (37 to 64%) of double-positive CD81^+^/CD9^+^ EVs in CD63-, CD81-, and CD9-captured spots have been found in the CSF (Figure 2C). Serum samples showed a high percentage (20 to 40%) of double-positive CD63^+^/CD9^+^ EVs in CD63-, CD9-, and CD41a-captured spots (Figure 2D). The next analysis was conducted considering all samples from both CSF and serum. A complete segregation between the tetraspanin pattern of CSF and serum was observed for CD63- (Figure S2A), CD81- (Figure S2B), and CD9-captured spots (Figure S2C).

### 3.3. GLAST^+^ EVs Exhibit a Lower Quantity in CSF from PPMS Compared to Both RRMS and Non-MS Subjects

**Figure 3:**
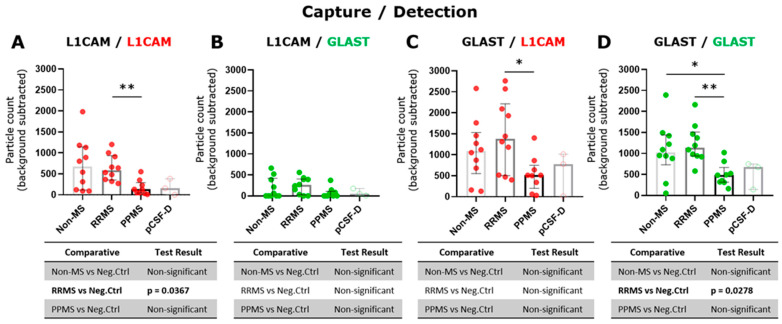
A higher L1CAM^+^ and GLAST^+^ EV levels were exhibited in CSF from RRMS compared to PPMS individuals. Single L1CAM^+^ EVs in (**A**) L1CAM- and (**C**) GLAST-captured spots and single GLAST^+^ EVs in (**B**) L1CAM- and (**D**) GLAST-captured spots measured in triplicate from CSF samples by ExoView R200+ technology. (**A**) A significant increase in the particle count of L1CAM^+^ EVs in RRMS compared to PPMS was observed (** *p* < 0.01; Brown–Forsythe and Welch ANOVA test, Games-Howell’s post test). (**C**) A significant increase in the particle count of L1CAM^+^ EVs in RRMS compared to PPMS was detected (* *p* < 0.05; ordinary one-way ANOVA test, Tukey’s post test). (**D**) A significant increase in the particle count of GLAST^+^ EVs in both RRMS and non-MS samples compared to PPMS was detected (** *p* < 0.01, * *p* < 0.05, respectively; ordinary one-way ANOVA test, Tukey’s post test). The particle count of the isotype control (IgG) was subtracted from the particle count on each capture spot. Negative controls of EV disrupted CSF samples (n = 3) were used to compare with the level of each fluorescent detection antibodies in intact biofluids. Data are expressed as the median ± IQR of at least nine independent determinations by ExoView R200+ technology. pCSF-D: pool of disrupted CSF samples.

**Figure 4:**
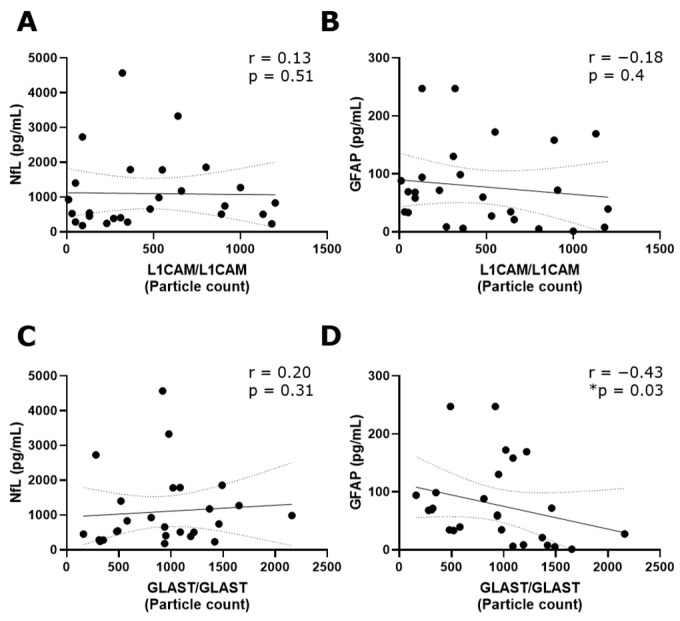
A negative and significant correlation was revealed between GLAST^+^ EVs and GFAP. Spearman correlation analysis revealed (**A**) a low positive correlation between L1CAM^+^ EVs in L1CAM-captured spots and NfL (r = 0.13) and (**B**) a low negative correlation with GFAP (r = −0.18). (**C**) A low positive correlation between GLAST^+^ EVs in GLAST-captured spots was observed with NfL (r = 0.20), while (**D**) a medium and statistically significant negative correlation was detected with GFAP (r = −0.43, * *p* < 0.05). These analyses were carried out considering all MS and non-MS samples. NfL and GFAP results were used in the correlation analysis as the average of triplicate values (measured in pg/mL) obtained for each sample using the commercial Ella™ microfluidic platform.

The tetraspanin flex chip enabled the study of CD63-, CD81-, CD9-, L1CAM- and GLAST-captured spots using L1CAM, GLAST, and CD9 as detection antibodies. Data that resulted in negative values after background subtraction were replaced with zero. In general, the obtained fluorescent signals were predominantly single, with colocalizations (double and triple signals) being insignificant in percentage. Considering this, the results were expressed as the addition of the signals for each fluorophore detected at each capture spot. In this context, GLAST-captured spots showed a significant increase in the number of L1CAM^+^ and GLAST^+^ EVs compared to L1CAM-captured spots (Figure S3). Then, the L1CAM^+^ and GLAST^+^ signals in L1CAM- and GLAST-captured spots were contrasted among RRMS, PPMS, and non-MS samples. Due to the basal background increase in customized flex chips, negative controls represented by disrupted EV samples were included into the analysis. Only L1CAM^+^ EVs in L1CAM-captured spots (Figure 3A) and GLAST^+^ EVs in GLAST-captured spots (Figure 3D) showed a significant increase in the particle count when intact RRMS samples were compared with disrupted EV samples in CSF. Moreover, a tendency toward a higher particle count of L1CAM^+^ EVs in GLAST-captured spots from intact RRMS and non-MS samples, and GLAST^+^ EVs in GLAST-captured spots from intact non-MS samples in comparison with disrupted EV samples, were observed. Interestingly, a significant decrease in the particle count of L1CAM^+^ EVs was detected in L1CAM-captured spots (Figure 3A), and L1CAM^+^ (Figure 3C) and GLAST^+^ EVs (Figure 3D) in GLAST-captured spots in the CSF from PPMS subjects compared to RRMS. Additionally, only GLAST^+^ EVs presented a significant decrease in CSF from PPMS compared to non-MS subjects. On the other hand, we studied the correlation of L1CAM^+^ EVs levels in L1CAM-captured spots and GLAST^+^ EVs levels in GLAST-captured spots from all groups with the NfL (axonal damage) [19,20] and GFAP (MS progression) established biomarkers [21]. A low positive correlation was observed between L1CAM^+^ EVs and NfL (r = 0.13; Figure 4A), and a low negative correlation with GFAP (r = −0.18; Figure 4B). In the case of GLAST^+^ EVs, a low positive correlation was observed with NfL (r = 0.20; Figure 4C), and a medium negative and significant correlation with GFAP (r = −0.43, *p* = 0.03; Figure 4D).

Considering tetraspanin-captured spots, neither CD63-, CD81-, nor CD9-captured spots, analyzing L1CAM^+^ and GLAST^+^ EVs, exhibited a significant difference between intact and disrupted EV samples in CSF (Figure S4). Similarly, neither L1CAM- nor GLAST-captured spots, analyzing CD9^+^ EVs, revealed a significant difference between intact and disrupted EV samples in CSF (Figure S5A,B). In contrast, CD9^+^ EVs in CD63-, CD81-, and CD9-captured spots displayed a significant increase between intact and disrupted EV samples in CSF (Figure S5C–E). However, CD9^+^ EVs in CD63-, CD81-, and CD9-captured spots did not show significant differences among non-MS, RRMS, and PPMS samples.

### 3.4. Neither L1CAM^+^ nor GLAST^+^ EV Levels Could Be Positively Revealed by ExoView Platform in Unprocessed Serum

The next objective was to analyze differences in L1CAM^+^ and GLAST^+^ EV levels between RRMS and PPMS individuals, mirroring those observed in CSF. However, neither L1CAM- nor GLAST-captured spots, when analyzed L1CAM^+^ and GLAST^+^ EVs, exhibited differences between intact and disrupted EV samples in serum (Figure S6). Similarly, when we analyzed CD9^+^ EVs in L1CAM- and GLAST-captured spots, neither L1CAM- nor GLAST-captured spots revealed a significant difference between intact and disrupted EV samples in serum (Figure S7A,B). In contrast, CD9^+^ EVs in CD63-, CD81-, and CD9-captured spots displayed a significant increase between intact and disrupted EV samples in serum. Furthermore, the particle count of CD9^+^ EVs in CD9-captured spots showed a significant increase in RRMS compared to PPMS.

## 4. Discussion

Tetraspanins are EV proteins widely studied using traditional methods that inevitably provide bulk results, averaging information from different EV subpopulations. However, single EV analysis is expected to reveal the precise EV composition, reflecting its specific biological diversity through its co-localization pattern [22,23]. In our data, the tetraspanin colocalization pattern did not allow us to differentiate RRMS, PPMS and non-MS donors neither in CSF nor serum. Instead, this pattern was only associated with the type of biofluid analyzed in both MS and non-MS individuals. Supporting this, Okada-Tsuchioka et al. [24] detected in completely human healthy biofluids that tetraspanins were specific to the sample type. Moreover, our MS and non-MS pathological samples displayed a significantly lower enrichment of CD63^+^ EVs in CSF and CD81^+^ EVs in serum, and a higher percentage of double-positive CD81^+^/CD9^+^ EVs in CSF and CD63^+^/CD9^+^ in serum. In line with results obtained from the healthy individuals [24] Although EVs displayed a complex tetraspanin pattern reflecting their cells of origin [25], the tracking of EVs using tetraspanins might not be useful to stratify MS with respect to other non-MS pathologies or between different MS subtypes, such as RRMS and PPMS, and may be irrelevant to obtain disease-specific information from the central nervous system (CNS).

Even less explored is one of the most interesting features of the ExoView platform: quantifying single EV levels with our proteins of interest. In MS, GLAST and L1CAM have been reported as captured proteins to enrich using plasma putative neuron- and astrocytes-derived EVs, respectively, using the ExoQuick methodology [14]. They reported that the concentration of GLAST^+^ EVs in plasma, as measured by nanoparticle tracking analysis, was higher in controls than in individuals with MS, while L1CAM^+^ EVs did not differ between them. In unprocessed CSF samples of our work, both L1CAM^+^ and GLAST^+^ EVs show almost no presence specifically in PPMS, being significantly different from RRMS. Additionally, only GLAST^+^ EVs exhibited a lower quantity in PPMS compared to non-MS samples. Altogether, our findings from CSF and previous plasma studies suggest that the concentration of GLAST^+^ EVs in MS samples, and specifically in PPMS, may show a consistent decline in both the CNS and periphery, supporting the hypothesis of their CNS origin. However, neither L1CAM^+^ nor GLAST^+^ EVs could be positively evidenced by the ExoView platform in our unprocessed serum samples. One possible explanation is that its low concentration, with respect to the high number of EVs from other tissues circulating in blood [26], is not sensitive enough to be detected with this platform, at least without an EV isolation method.

On the other hand, our experiments suggest a lower presence of L1CAM^+^ EVs compared to GLAST^+^ EVs in CSF (Figure S3), at least under the studied conditions, since GLAST-captured spots showed more EVs in both L1CAM and GLAST detection. The differences between capturing with L1CAM or GLAST and detecting with the others (Figure 3B,C) highlight the importance of the capture antibody in this technique, which must be considered in further analysis. Moreover, L1CAM- and GLAST-derived EVs showed a very low presence of tetraspanin proteins. This finding suggests that studies of CNS-derived EVs might utilize both capture and detection antibodies targeting the same protein, because the use of only tetraspanins as the captured antibodies could lead us to lose relevant information.

To gain a deeper biological understanding of our findings, a correlation analysis with established neural biomarkers was performed. This suggests that lower levels of GLAST+ EVs were correlated with an increase in GFAP levels in CSF. Intriguingly, the presence of GFAP in biofluids—the main intermediate filament protein within astrocytes—is associated with astrocyte damage and the presence of neurodegenerative processes [27], consistent with the worst clinical status of PPMS vs. RRMS (EDSS 4 vs. 1.25). In MS, GFAP reflects chronic astrocyte-mediated disease processes that manifest as MS progression independent of relapse activity, different to NfL, which is associated with neuronal damage during acute disease activity [21]. Furthermore, GLAST is the glutamate aspartate transporter localized in the astrocyte cell membrane and the inner mitochondrial membrane, mediating the transport of glutamic and aspartatic acid. The importance of glutamate homeostasis and the prevention of glutamate excitotoxicity has been reported in MS and other neurodegenerative diseases [28,29,30,31,32]. Summarizing, these results could propose an astrocyte dysfunction specifically in PPMS, with a lower production of these EVs, and a relation with glutamate metabolism. 

The selected samples are diagnostic, meaning that the clinical symptoms have appeared relatively near to their obtention. Given that MS samples present an OCB-IgG pattern in CSF but not in serum, pwMS is showing a neural inflammatory process. In this sense, the differences observed in the L1CAM^+^ and GLAST^+^ EVs in CSF between MS samples highlight the different molecular mechanisms involved from the beginning in the etiology of PPMS and RRMS. Moreover, they could shed light on whether these MS subtypes are different diseases or the same disease with different degrees of aggressiveness. Further studies in bigger series would help to expand on these findings, considering that this research related to CNS-EV biomarkers is not only useful for diagnostic clinical outcomes but also provides a window to uncover mechanisms underlying the PPMS disease. Furthermore, given that the age of the PPMS group is always higher than RRMS group [17], the age influence in our findings remains elusive.

## 5. Conclusions

This is the first study in which single tetraspanins, L1CAM^+^ and GLAST^+^ EVs were tracked in unprocessed biofluids as biomarkers from pwMS. ExoView technology could be useful to track CNS-derived EV biomarkers and be potentially valuable in the diagnostic evaluation and follow-up of pwMS. GLAST^+^ EVs might provide insights into the etiology of PPMS and could offer small windows to elucidate the molecular mechanisms behind its clinical presentation.

## Figures and Tables

**Table 1:** Characteristics of the patients whose CSF and serum samples were utilized in this study.

Type	Age (Years) Mean ± SD	Sex (F/M)	EDSS Median (IQR)	n
Non-MS	45 ± 16	8/2	-	10
RRMS	36 ± 13	8/2	1.25 (0–3)	10
PPMS	51 ± 8	7/2	4 (4–5.9)	9

EDSS: expanded disability status scale.

## Data Availability

All analyzed data during this study are included in this article and its Supplementary Information Files. The obtained ExoView R200+ raw data and the scripts developed in-house to analyze these data will be provided to any interested investigators upon request and without undue reservation via email.

## Supplementary Materials

The following supporting information can be downloaded at: https://www.mdpi.com/article/10.3390/biomedicines12102245/s1, Figure S1. Comparison of total particle count between intact and disrupted EV samples; Figure S2. Tetraspanin colocalization pattern is specifically linked to the type of biofluid analysed; Figure S3. GLAST-captured spots revealed a higher quantity of both L1CAM+ and GLAST+ EVs in CSF; Figure S4. L1CAM+ and GLAST+ EVs showed a low presence of tetraspanins; Figure S5. CD9+ EVs showed a low presence of CNS-derived EV proteins; Figure S6. Neither L1CAM+ nor GLAST+ EV levels could positively revealed by ExoView platform in unprocessed serum; Figure S7. CD9-captured spots revealed a higher quantity of CD9+ EVs in serum.

## Abbreviations

CNS	central nervous system
CSF	cerebrospinal fluid
GFAP	glial fibrillary acidic protein
GLAST	glutamate aspartate transporter
IQR	interquartile range
L1CAM	L1 cell adhesion molecule
MS	multiple sclerosis
NfL	neurofilament light
OCB	oligoclonal bands
PPMS	primary progressive multiple sclerosis
pwMS	people with multiple sclerosis
RRMS	relapsing–remitting multiple sclerosis
RT	room temperature
SP-IRIS	single-particle interferometric reflectance imaging sensor
