# Supplementary material for: Tetraspanins, GLAST and L1CAM Quantification in Single Extracellular Vesicles from Cerebrospinal Fluid and Serum of People with Multiple Sclerosis
Source: Biomedicines. 2024 Oct 2;12(10):2245. doi: 10.3390/biomedicines12102245 (PMC11504864; doi:10.3390/biomedicines12102245)
Supplement: Supplementary file 1 [file biomedicines-12-02245-s001.zip › biomedicines-3220039-Supplementary.pdf]

Supplementary Figures and Legends

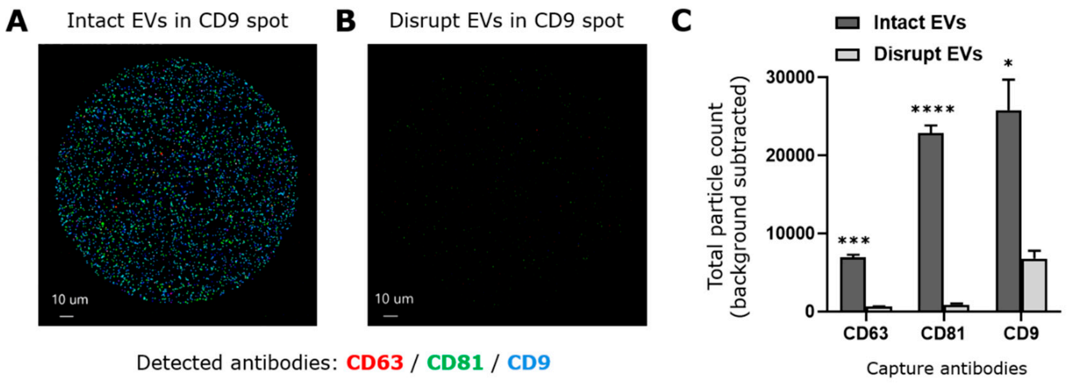

Supplementary Figure S1. Comparison of total particle count between intact and disrupted EV samples

Representative SP-IRIS-based ExoView R200+ images of CD9-captured spot incubated with a pool of **(A)** intact and **(B)** disrupted CSF sample. **(C)** Total single EV captured (CD63-spots, CD81-spots, CD9-spots) measured in triplicate from CSF samples. A significant increase in the total particle count of intact vs disrupted EV in CD63-, CD81, and CD9-captured spots was observed (***p < 0.001, ****p < 0.0001, *p < 0.05, respectively; unpaired t test). The total particle count of the isotype control (IgG) was subtracted from the total particle count on each capture spot. Data are expressed as the median ± range of a pool from the three types of samples used in the study, measured using ExoView R200+ technology.

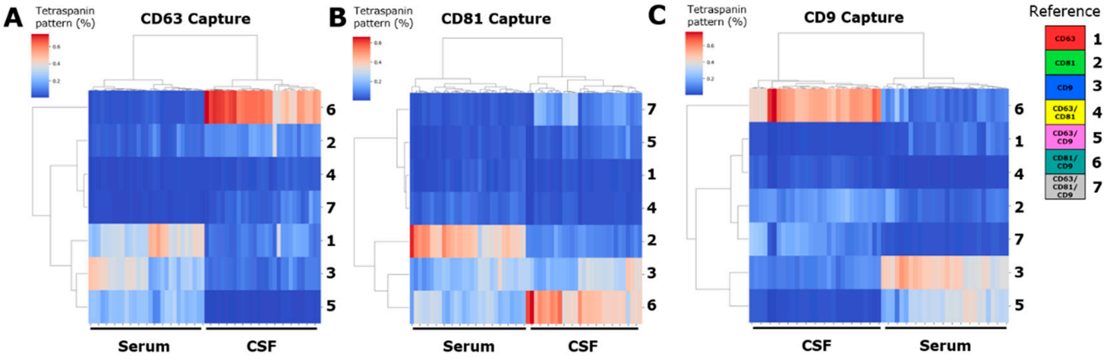

Supplementary Figure S2. Tetraspanin colocalization pattern is specifically linked to the type of biofluid analysed

Unsupervised hierarchical clustering of the tetraspanin colocalization pattern in **(A)** CD63-spots, **(B)** CD81-spots, **(C)** CD9-spots in CSF vs serum from MS and non-MS individuals. The tetraspanin pattern achieves a discrimination between the two biofluids in all analysed

captured spots. Data are expressed as the percentage of at least nine independent determinations by ExoView R200+ technology. To note, the CD41a platelet marker was analysed only in serum, thus CD41a-captured spots were not included in this analysis.

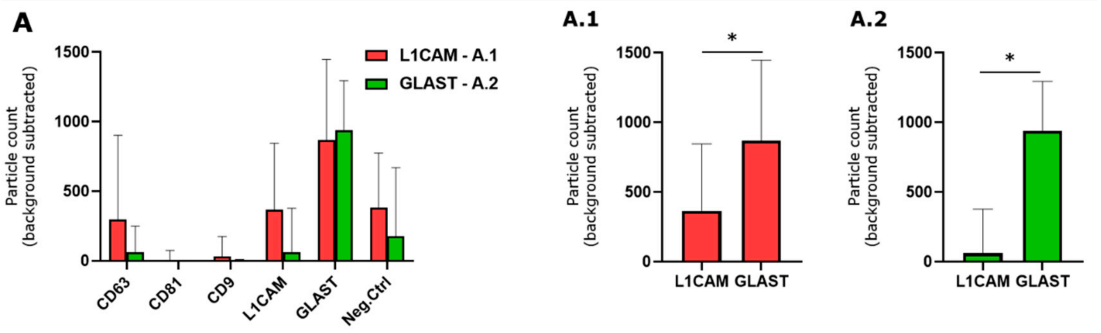

Supplementary Figure S3. GLAST-captured spots revealed a higher quantity of both L1CAM$^+$ and GLAST$^+$ EVs in CSF

(A) Single L1CAM$^+$ and GLAST$^+$ EVs in CD63-, CD81-, CD9-, L1CAM-, and GLAST-captured spots in CSF. **(A.1)** A significant increase in the particle count of L1CAM$^+$ EVs in GLAST-captured spots compared to L1CAM-captured spots was detected (**$p < 0.01$; unpaired Mann Whitney test). **(A.2)** A significant increase in the particle count of GLAST$^+$ EVs in GLAST-captured spots compared to L1CAM-captured spots was observed (****$p < 0.0001$; unpaired Mann Whitney test). Data are expressed as the median ± IQR of at least nine independent determinations by ExoView R200+ technology.

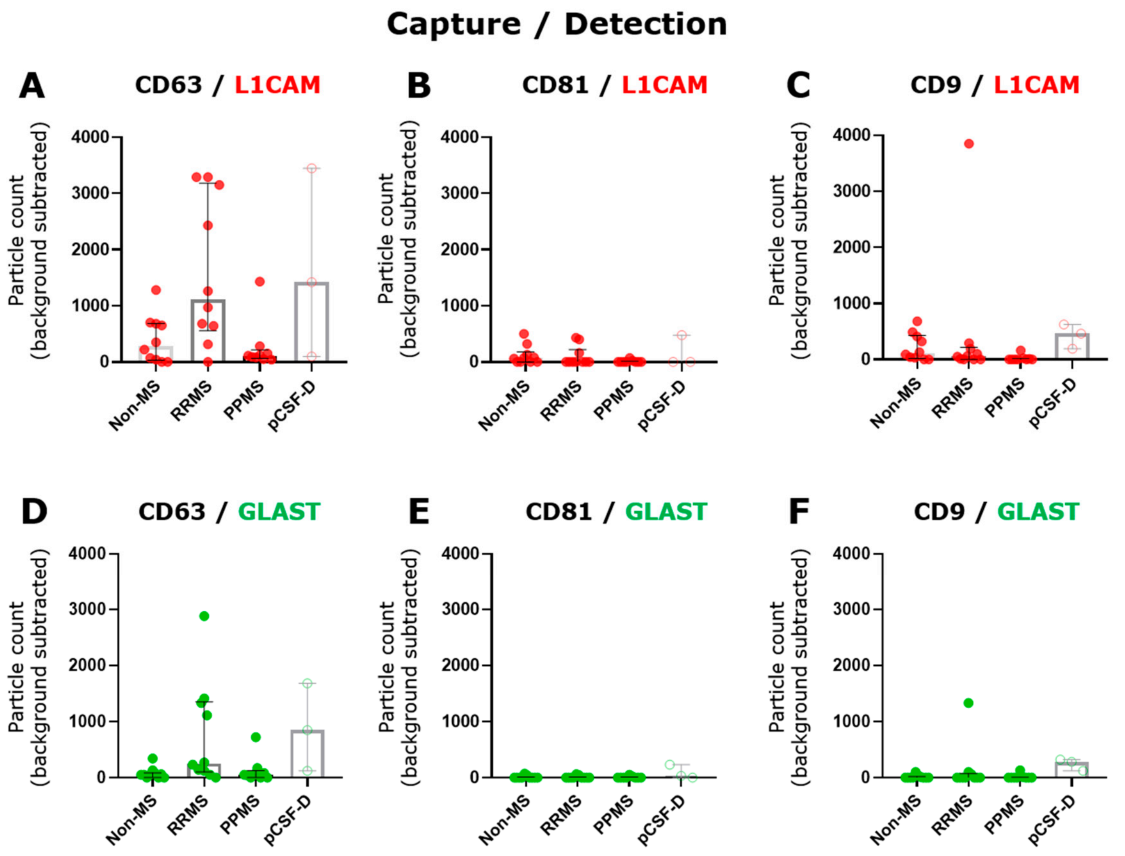

Supplementary Figure S4. L1CAM⁺ and GLAST⁺ EVs showed a low presence of tetraspanins

Single L1CAM⁺ EVs in **(A)** CD63-, **(B)** CD81-, and **(C)** CD9-captured spots measured in triplicate from CSF samples. Single GLAST⁺ EVs in **(D)** CD63-, **(E)** CD81-, and **(F)** CD9-captured spots measured in triplicate from CSF samples. The particle count of the isotype control (IgG) was subtracted from the particle count on each capture spot. Negative controls of EV disrupted CSF samples (n=3) were used to compare with the level of each fluorescent detection antibodies in intact biofluids. Data are expressed as the median ± IQR of at least nine independent determinations by ExoView R200+ technology. pCSF-D: pool of disrupted CSF samples.

Capture / Detection

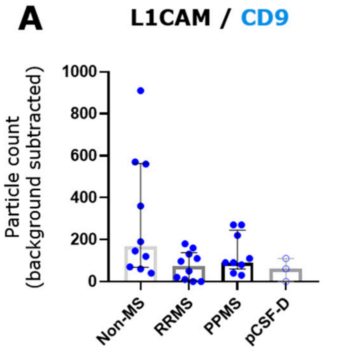

Comparative	Test Result
Non-MS vs Neg.Ctrl	Non-significant
RRMS vs Neg.Ctrl	Non-significant
PPMS vs Neg.Ctrl	Non-significant

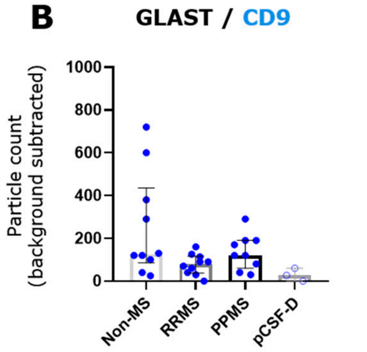

Comparative	Test Result
Non-MS vs Neg.Ctrl	Non-significant
RRMS vs Neg.Ctrl	Non-significant
PPMS vs Neg.Ctrl	0,0574

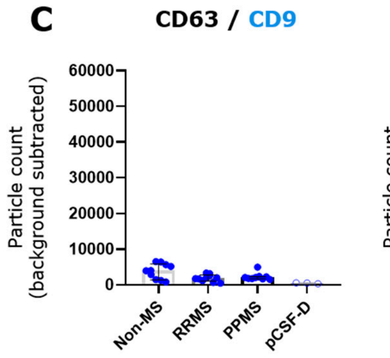

Comparative	Test Result
**Non-MS vs Neg.Ctrl**	**p = 0,0231**
**RRMS vs Neg.Ctrl**	**p = 0,0306**
**PPMS vs Neg.Ctrl**	**p = 0,0091**

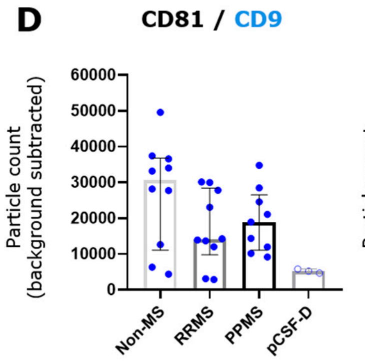

Comparative	Test Result
**Non-MS vs Neg.Ctrl**	**p = 0,0303**
RRMS vs Neg.Ctrl	p = 0,07
**PPMS vs Neg.Ctrl**	**p = 0,0233**

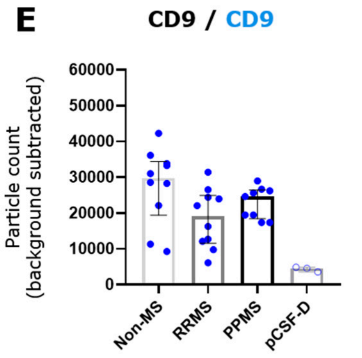

Comparative	Test Result
**Non-MS vs Neg.Ctrl**	**p = 0,0035**
**RRMS vs Neg.Ctrl**	**p = 0,0152**
**PPMS vs Neg.Ctrl**	**p < 0,0001**

Supplementary Figure S5. CD9^{+} EVs showed a low presence of CNS-derived EV proteins

Single CD9^{+} EVs in **(A)** L1CAM-, **(B)** GLAST-, **(C)** CD63, **(A)** CD81-, and **(B)** CD9-captured spots measured in triplicate from CSF samples. The particle count of the isotype control (IgG) was subtracted from the particle count on each capture spot. Negative controls of EV disrupted CSF samples (n=3) were used to compare with the level of each fluorescent detection antibodies in intact biofluids. Data are expressed as the median ± IQR of at least nine independent determinations by ExoView R200+ technology. pCSF-D: pool of disrupted CSF samples.

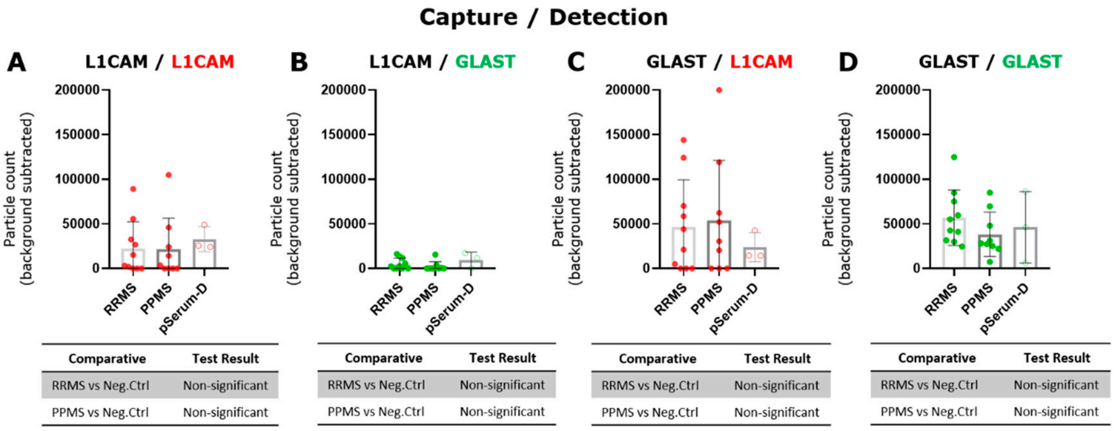

Supplementary Figure S6. Neither L1CAM$^+$ nor GLAST$^+$ EV levels could positively revealed by ExoView platform in unprocessed serum

Single L1CAM$^+$ EVs in **(A)** L1CAM- and **(C)** GLAST-captured spots and single GLAST$^+$ EVs in **(B)** L1CAM- and **(D)** GLAST-captured spots measured in triplicate from serum samples by ExoView R200+ technology. The particle count of the isotype control (IgG) was subtracted from the particle count on each capture spot. Negative controls of EV disrupted serum samples (n=3) were used to compare with the level of each fluorescent detection antibodies in intact biofluids. Data are expressed as the median ± IQR of at least nine independent determinations by ExoView R200+ technology. pSerum-D: pool of disrupted serum samples.

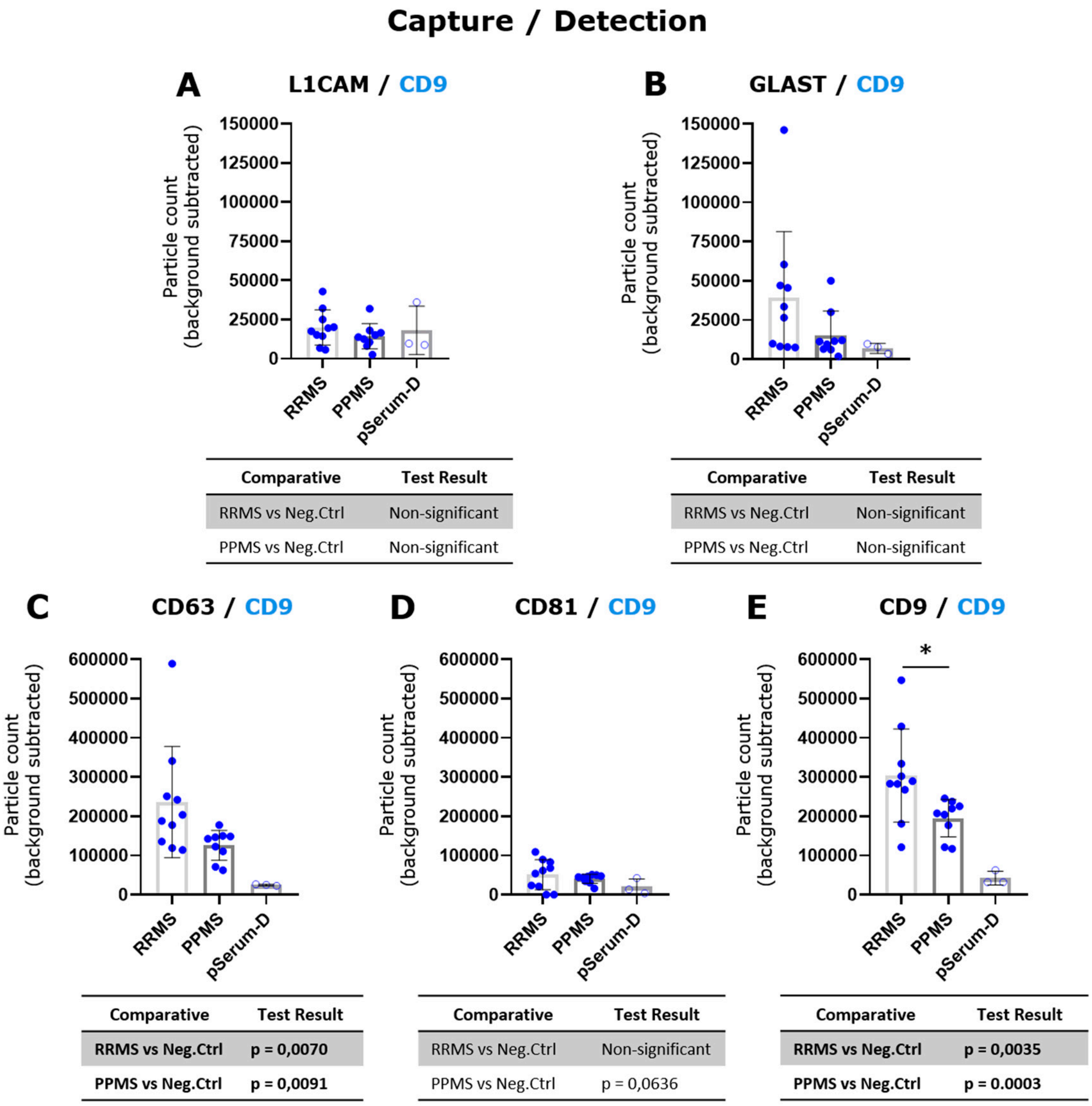

A

Comparative	Test Result
RRMS vs Neg.Ctrl	Non-significant
PPMS vs Neg.Ctrl	Non-significant

B

Comparative	Test Result
RRMS vs Neg.Ctrl	Non-significant
PPMS vs Neg.Ctrl	Non-significant

C

Comparative	Test Result
**RRMS vs Neg.Ctrl**	**p = 0,0070**
**PPMS vs Neg.Ctrl**	**p = 0,0091**

D

Comparative	Test Result
RRMS vs Neg.Ctrl	Non-significant
PPMS vs Neg.Ctrl	p = 0,0636

E

Comparative	Test Result
**RRMS vs Neg.Ctrl**	**p = 0,0035**
**PPMS vs Neg.Ctrl**	**p = 0.0003**

Supplementary Figure S7. CD9-captured spots revealed a higher quantity of $CD9^+$ EVs in serum

Single $CD9^+$ EVs in **(A)** L1CAM-, **(B)** GLAST-, **(C)** CD63-, **(D)** CD81-, and **(E)** CD9-captured spots measured in triplicate from CSF samples. **(E)** A significant increase in the particle count of $CD9^+$ EVs in RRMS compared to PPMS was detected (*$p < 0.05$; unpaired t test). The particle count of the isotype control (IgG) was subtracted from the particle count on each capture spot. Negative controls of EV disrupted serum samples (n=3) were used to compare with the level of each fluorescent detection antibodies in intact biofluids. Data are expressed as the median ± IQR of at least nine independent determinations by ExoView R200+ technology. pSerum-D: pool of disrupted serum samples.
